# Supplementary material for: Effect of Genetic Variability in 20 Pharmacogenes on Concentrations of Tamoxifen and Its Metabolites
Source: J Pers Med. 2021 Jun 4;11(6):507. doi: 10.3390/jpm11060507 (PMC8228634; doi:10.3390/jpm11060507)
Supplement: Supplementary file 1 [file jpm-11-00507-s001.zip › jpm-1228893-supplementary.pdf]

Supplementary Tables:

Supplementary Table 1: Allelic Activity Designations. *CYP2D6* adapted from CPIC guideline [1].

Genes	Low Activity	Normal Activity	High Activity
CYP2D6	CPIC guidance	CPIC guidance	CPIC guidance
CYP3A4	*20,*22	*1	
CYP3A5	*3	*1	
ABCB1	rs1045642	WT	
SLCO1B1	*5 (rs4149056)	*1	
CYP1A2	*1C (rs2069514), *1K (rs762551, rs12720461), *1FC (rs2069514, rs762551)	*1	*1F (rs762551)
CYP2B6	*6, *13, *18	*1, *2	
CYP2C9	*2, *5, *6, *8, *11, *12 (AS = 0.5) *3 (AS = 0)	*1, *9	
CYP2C19	*2, *3, *8	*1	*17
APOE	E2	E3, E4	
COMT	rs4680	*1	
DRD2	rs1800497	*1	
F2	rs1799963	*1	
F5	rs6025	*1	
GLP1R	rs1042044, rs6923761	*1, rs2300615	
MTHFR	rs1801133	*1, rs1801131	
OPRM1	rs1799971	*1	
PNPLA5	rs5764010	*1	
SULT4A1	rs76312	*1	
VKORC1	*2 (rs9923231)	*1	

Supplementary Table 2: Phenotype Activity Designations. *CYP2D6* adapted from CPIC guideline [1]

Genes	Poor	Intermediate	Normal	Rapid	Ultra-rapid
CYP2D6	CPIC guidance	CPIC guidance	CPIC guidance		CPIC guidance
CYP3A4	L/L	N/L	N/N		
CYP3A5	L/L	N/L	N/N		
ABCB1	L/L	N/L	N/N		
SLCO1B1	L/L	N/L	N/N		
CYP1A2	L/L	N/L or H/L	N/N		N/H or H/H
CYP2B6	L/L	N/L	N/N		
CYP2C9	L/L	N/L	N/N		
CYP2C19	L/L	N/L or H/L	N/N	N/H	H/H
APOE	L/L	N/L	N/N		
COMT	L/L	N/L	N/N		
DRD2	L/L	N/L	N/N		
F2	L/L	N/L	N/N		
F5	L/L	N/L	N/N		
GLP1R	L/L	N/L	N/N		
MTHFR	L/L	N/L	N/N		
OPRM1	L/L	N/L	N/N		
PNPLA5	L/L	N/L	N/N		
SULT4A1	L/L	N/L	N/N		
VKORC1	L/L	N/L	N/N		
